# Relationship between Duration of Fluoride Exposure in School-Based Fluoride Mouthrinsing and Effects on Prevention and Control of Dental Caries

**DOI:** 10.5402/2012/183272

**Published:** 2012-04-04

**Authors:** Eri Komiyama, Kazunari Kimoto, Hirohisa Arakawa

**Affiliations:** Division of Oral Health, Department of Health Science, Kanagawa Dental College, 82 Inaoka-cho, Yokosuka, Kanagawa 238-8580, Japan

## Abstract

The objective of this paper was to assess the effects of school-based fluoride mouthrinsing (S-FMR: weekly using 0.2% NaF solution) in two groups of school children with different periods of exposure to S-FMR in elementary school. Subjects were the S-FMR group consisted of 599 children, participated for six years. The control group consisted of 282 children, participated for less than one year in the sixth year of elementary school. From the results of the present survey, the caries reduction rate of S-FMR in the permanent teeth was 36.6% for DMFT and 42.8% for DMFS, and person rates with DMF, DMFT, DMFS, and CO (questionable caries under observation) were inhibited in both boys and girls. Girls in the control group showed clearly higher values for all parameters of dental caries because of earlier teeth eruption; however, no gender differences were observed in the S-FMR group. As caries prevalence in the first molars accounted for about 85% regardless of participation to S-FMR, and first molar caries were more common in the mandible than in the maxilla, consideration should be given to preventive measures against pit-and-fissure-caries in addition to S-FMR.

## 1. Introduction

In Japan, where methods for systemic fluoride application such as water fluoridation have not been performed, school-based fluoride mouthrinsing started in the 1970s and many successful results have been reported [1–6]. Although fluoride mouthrinsing is one of topical fluoride application, it has many advantages: it can be performed at one time in groups; it has reliable preventive effects; it is safe; it is simple to apply; it has excellent cost benefits; it allows for continuous group application [7–14]. Therefore, as fluoride mouthrinsing is of great importance to public health, it should be provided via group application in Japan [1, 2, 15–17]. In Japan, school-based fluoride mouthrinsing (S-FMR) started in one prefecture in 1970 and had expanded to all 47 prefectures by 2005 [3]. According to latest nationwide survey performed by the Nonprofit Japanese Conference on the Promotion of the Use of Fluoride in Caries Prevention (NPO-JPUF), the 8020 Promotion Foundation, and the WHO Collaborating Center for Translation of Oral Health Science (WHOCC Niigata), S-FMR is performed in 7,479 schools by 777,596 children nationwide, as of the end of March 2010, which corresponds to about 6% of 4- to 14-year-old children in Japan [17]. The number of persons undergoing fluoride mouthrinsing worldwide in 2000, including both S-FMR and home application, was estimated to be 100 million [18]. 

The S-FMR program in the present study began as a model project in two elementary schools and one junior high school in the targeted municipality in 1971, and by 2005, it had expanded to all 19 elementary schools and all seven junior high schools in the municipality. In this study, nine elementary schools and three junior high schools participated in S-FMR for the first time. S-FMR started in May and June 2005 in three elementary schools and one junior high school, and in September and October 2005 in six elementary schools and two junior high schools, as a result of differences in the preparatory periods for introduction of S-FMR in each school. In Japan, the school year runs from April to March of the following year. Therefore, from a comparison of dental caries prevalence in first-year students entering all seven junior high schools in 2006 from the nine elementary schools participating from 2005, and the 10 elementary schools previously participating in S-FMR, it was possible to judge the efficacy of S-FMR. We examined 12 year olds (first-year of junior high school; grade seven) in the scheduled spring dental examination based on the School Health Law of Japan in April 2006 and surveyed the prevalence of dental caries. The objective of this paper was to assess the effects of S-FMR in two groups of school children with different periods of exposure to S-FMR in elementary school.

## 2. Subjects and Methods

### 2.1. Children Subjected to Survey

Examinations were performed in the scheduled spring dental examinations, based on the School Health Law, for 1,005 first-year students in all seven municipal junior high schools. When children who were absent from school on the date of the examination and children who had transferred from other elementary schools were excluded, the S-FMR exposure group (F+ group) consisted of 599 children (boys 319, girls 280), and the group not exposed to S-FMR (F− group) consisted of 282 children (boys 147, girls 135). The F+ group had participated in S-FMR for six years in ten elementary schools, while the F− group had participated in S-FMR for less than one year in the sixth-year of nine elementary schools (S-FMR in three schools started from May or June, and S-FMR in six schools started from September or October). S-FMR in elementary school consisted of fluoride mouthrinse for 60 seconds once a week using 10 mL of 0.2% sodium fluoride solution, containing 900 ppm fluoride.

For the protection of privacy, compliance with the Personal Information Protection Law was assured, and survey forms for the subjects used random numbers to indicate gender and S-FMR experience (elementary school attended). The survey was performed in compliance with the Ethical Policies for Epidemiological Research of the Ministry of Health, Labor and Welfare and the Helsinki Declaration, and the results were computed based on relevant ethical considerations (Notification number 40 of the approval of the Ethics Committee of Kanagawa Dental College).

### 2.2. Examiners

The examiners were three dentists in the Division of Oral Health, Department of Health Science, Kanagawa Dental College. These three dentists had undergone adequate training for this examination and performed calibration before the date of examination. The agreement rate (*κ* value) for caries diagnosis among the examiners conducted several times using extracted teeth was not less than 0.8. Information on whether the subjects had participated in S-FMR was not given to the examiners.

### 2.3. Contents of Examination

The examination was performed based on tooth surface units using visual dental caries detection by WHO standards [19]. Dental caries examination standards used in this survey were based on the contents of examinations according to the School Health Law, and the examination details for each child were transferred to the student health records (dental/oral cavity) after examination. The contents of the examination used for analyses in this survey were as follows: (1) current permanent teeth; (2) dental caries: permanent teeth were examined for caries by tooth or tooth surface units; and (3) questionable caries under observation (CO), for example, white spot lesion, without dental caries lesion on visual. Examination was based on the Enforcement Regulations of the School Health Law of Japan (amended in 1995). In Japan, CO is diagnosed as a sound tooth, “0” under WHO code [19].

### 2.4. Method of Calculation and Account

Calculations and accounts for dental caries in evaluation of the effects of S-FMR were performed only on permanent teeth. Using the decayed-missing-filled (DMF) caries status, the DMF incidence, and mean DT-DS, MT-MS, FT-FS, DMFT, and DMFS (T: tooth; S: tooth surface) are shown for each subject. DMFT and DMFS were also calculated for specific dentition and groups of teeth. For CO teeth, the person rate of CO, mean CO number and percentage of CO teeth among total teeth, and mean CO tooth surface number and percentage of CO tooth surfaces among total tooth surfaces were calculated. As CO teeth are not considered to have dental caries, they were not included in the totals for untreated teeth or untreated tooth surfaces (DT, DS). The numbers of CO teeth or tooth surfaces were shown as percentages of the total number of teeth and total number of tooth surfaces as indices for assessing the effects of S-FMR. The caries reduction rate of S-FMR was calculated using the total values of the F+ and F− groups for the elementary school that the children attended. It was assumed that children showed no differences in dental caries in the permanent teeth at the time of entering each elementary school. Therefore, the results were obtained by compiling (1), (2), and (3) previously.

For the prefectural mean value in the targeted municipality, the status of 12 year olds (total number surveyed: 20,867 first-year junior high school students) in the 2006 prefectural survey on school health statistics was used as a reference. The prefectural mean estimate was 1.4 (1.3 for boys and 1.5 for girls). For the nationwide value, the status of 12 year olds (sampling survey: about 284,000 first-grade year junior high school students) in the 2006 National survey on school health statistics of the Ministry of Education, Culture, Sports, Science and Technology was used as a reference. According to this report, the number of untreated teeth was 0.60 (0.57 for boys and 0.63 for girls), the number of missing teeth was 0.03 (0.02 for boys and 0.03 for girls), the number of treated teeth was 1.08 (0.98 for boys and 1.18 for girls), and DNFT index was 1.71 (1.57 for boys and 1.85 for girls). However, as these values include cases treated due to causes other than dental caries (untreated teeth, treated teeth, and missing teeth due to traumatic injury, missing teeth due to orthodontics, etc.), the values are given as reference.

### 2.5. Statistical Analysis

Excel was used for calculations. Statistical analysis of the results was performed by chi-squared test and Wilcoxon's rank sum test.

## 3. Results

### 3.1. Prevalence of Caries in Permanent Teeth

Table 1 shows the prevalence of dental caries in permanent teeth in terms of teeth and tooth surfaces by gender for 12 year olds (first-year of junior high school) in the two groups. The person rate with DMF was 46.1% in the F+ group and 64.9% in the F− group, which represented a statistically significant difference (*P* < 0.05). Particularly in the F− group, the prevalence was significantly higher in girls (71.9%) than that in boys (58.5%) (*P* < 0.05). In the F+ group, a difference between boys and girls was not observed. The DMFT index was 1.28 in the F+ group and 2.02 in the F− group, and the DMFS index was 2.05 in the F+ group and 3.69 in the F− group. Values in the F− group were significantly higher (*P* < 0.05). No gender differences were seen in the F+ group. However, in the F− group, DT, FT, DS, and FS were clearly higher in girls. In particular, the person rate with DMF, DMFT index, and DMFS index in both boys, and girls decreased in S-FMR. Eruption of permanent teeth was earlier in girls than in boys and the preventive effects of S-FMR were greater in girls, with about one more erupted tooth. In comparison with the prefectural mean value of 1.4, the value in the F+ group was lower. In comparison with the nationwide value of 1.71, the value in the F+ group was lower, but the value in the F− group was higher. In particular, the DT in the F+ group was lower than the nationwide value of 1/7, which was a good result, but the FT tended to be slightly higher. The caries reduction rate of S-FMR in the permanent teeth showed lower values of 36.6% for DMFT and 42.8% for DMFS.

**Figure 1:**
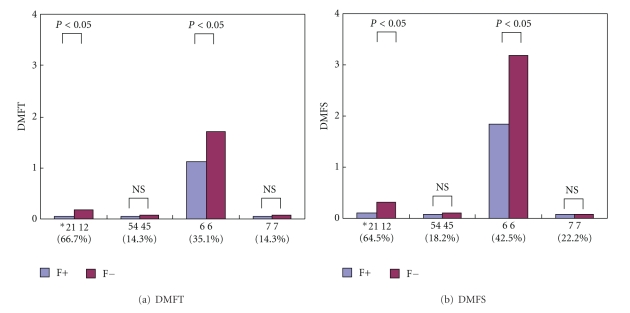
Comparison of DMFT and DMFS index by dentition between two groups. *21 12: Upper and lower central and lateral incisors; 54 45: Upper and lower first and second premolars; 6 6: Upper and lower first molars; 7 7: Upper and lower second molars; ( ) Caries reduction rate in dentition of permanent teeth after fluoride mouthrinsing program (%); NS: Not significant.

Table 2 shows the status of the teeth and tooth surface units by dentition of permanent teeth. For mean DMFT and DMFS by dentition of teeth, values were about the same in the F+ and F− groups for the premolars that erupted during the middle years of elementary school and the second molars that erupted from the sixth grade. However, in the first molars, central incisors, and lateral incisors that erupted before attending school or in the early grade years of elementary school, the values were significantly lower in the F+ group than those in the F− group (*P* < 0.05). No gender differences in dental caries were observed in the F+ group, and mean values of DT, FT, DS, and FS were clearly higher in girls in the F− group. The caries reduction rate in the F+ group showed a DMFT of 66.7% and DMFS of 64.5% for the central and lateral incisors. For first molars, DMFT was 35.1% and DMFS was 42.5%. Figure 1 shows the caries reduction rates for teeth with early eruption, and for premolars and second molars that were not markedly affected by fluoride mouth rinsing in elementary school, as the time of eruption was late. For teeth with late eruption, the value for the F+ group was slightly lower than that for the F− group, but the difference was not marked. However, in teeth with early eruption, high caries reduction rates of 35.1% to 66.7% were observed (*P* < 0.05). About 85% of caries in permanent teeth occurred in the first molars with no relation to participation in S-FMR.

Although not shown in the table, the effects of fluoride mouthrinsing on caries status in the first molars were examined. As no gender differences were seen in the F+ group, the totals for both genders were calculated. No differences between the right and left sides were seen, but the maxillary mean DMFT was 0.42 in the F+ group and 0.67 in the F− group, while the mandibular mean DMFT was 0.68 in the F+ group and 1.00 in the F− group. The maxillary mean DMFS was 0.69 in the F+ group and 1.32 in the F− group, and the mandibular mean DMFS was 1.14 in the F+ group and 1.86 in the F− group. However, no significant differences were observed between the prevalence of dental caries in the first molars in the maxilla and mandible. In the mean DMFS of the first molars, the percentages for tooth surfaces that experienced high levels of caries were 60.9% in the occlusal plane of the maxilla, 26.1% in the palatal plane, 57.9% in the occlusal plane of the mandible, and 29.8% in the buccal plane in the F+ group, as compared to 52.3% in the occlusal plane of the maxilla, 28.0% in the palatal plane, 53.8% in the occlusal plane of the mandible, and 25.8% in the buccal plane in the F− group.

### 3.2. Findings for CO

Findings for teeth and tooth surfaces of CO are shown in Table 3. The person rate with CO, mean CO number and CO teeth percentage per total teeth, and mean CO tooth surface number and CO tooth surface percentage per total tooth surfaces were significantly higher by 1.5-fold, 1.9-fold, 1.9-fold, 2.6-fold, and 2.7-fold, respectively, in the F− group than those in the F+ group (*P* < 0.001). For the CO teeth percentage per total teeth and CO tooth surface percentage per total tooth surfaces, the percentages with findings were 1.9-fold and 2.7-fold higher, respectively, in the F− group than in the F+ group. Similarly to caries prevalence, mean CO number and CO teeth percentage per total teeth and mean CO tooth surface number and CO tooth surface percentage per total tooth surfaces showed no gender differences in the F+ group.

**Figure 2:**
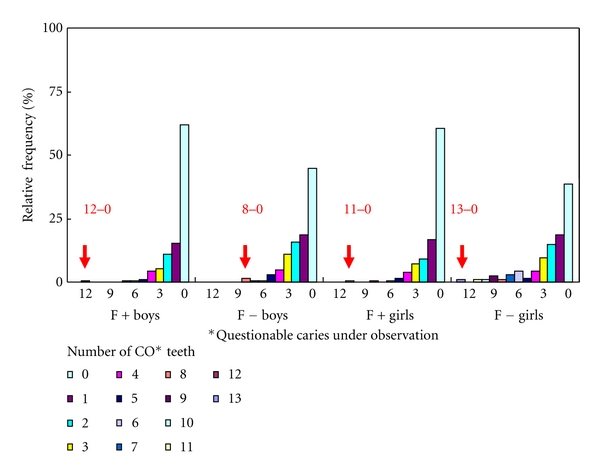
Relative frequency in number of CO* permanent teeth in two groups.

**Figure 3:**
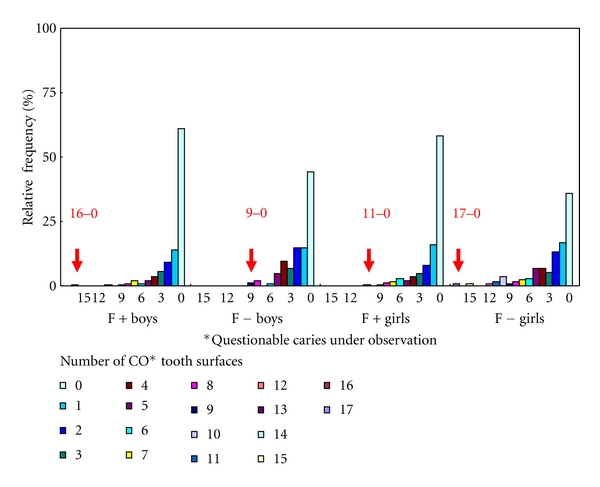
Relative frequency in number of CO* permanent tooth surfaces in two groups.

Therefore, for an investigation of future directions in aftercare and dental health guidance, the relative frequency in the CO tooth number and CO tooth surface number in subjects with CO teeth are shown in Figures 2 and 3. No CO gender differences were noted under S-FMR management, but there were high-risk students with numerous CO teeth or CO tooth surfaces. For high-risk students, the same cumulative curves were seen for both CO teeth and CO tooth surfaces.

## 4. Discussion

In relation to the application of fluoride in recent years, there have been opinions and announcements made by related organizations, including the Ministry of Health, Labor and Welfare, the Japan Dental Association, the Japanese Association for Dental Science, Japanese Association of School Dentists, the Japanese Society for Dental Health, the Japanese Society for Disability and Oral Health, and the Japanese Society of Pediatric Dentistry, and fluoride mouthrinsing has been recommended [2, 3, 16, 17, 20]. After the publication of the Inquiry of the Japanese Association for Dental Science, “Overall Opinion on Use of Fluoride” (1999), and “Guidelines on Fluoride Mouthrinse” (2003) by the Ministry of Health, Labor and Welfare, S-FMR spread to all prefectures in 2005. From 2000, as part of “Healthy Japan 21”, a movement to assure health that is being promoted generally and effectively by all health-related organizations and groups together with the general public, basic target values to be achieved by 2010 were established in order to assure longer, healthier lives. Measures are also being taken to raise public awareness concerning health. For dental health, the goal is to achieve “a nationwide mean DMFT 1 or less in 12 year olds” by 2010 [3, 16, 17, 20]. In 2006, the interim assessment results were proposed for “Healthy Japan 21” and instructions for fluoride mouthrinsing were specified as “a topic to be given priority in the future and for which new policies should be drafted” in “Topics to be Dealt with in the Future, 6. Dental Health” further dissemination of fluoride mouthrinsing is expected in the future [5]. In addition, for preschool children in Japan, the safety of fluoride mouthrinsing has been reported to be higher than that in Western countries, and on the WHO webpage “WHO Bank of Ideas,” it stated that in regions such as Japan, where there was no water fluoridation, the significance of performing S-FMR at the institutional level before children attend school was high [21, 22].

There have been many reports on the caries inhibitory effects of S-FMR to date in Japan, but there have been no assessments of caries inhibitory effects with blinded examiners in first-year junior high school students divided into a group exposed to S-FMR in six years of elementary school and a group exposed for less than one year in the sixth-year of elementary school, as in the present study. If the carries inhibitory effects of S-FMR can be proven by utilizing this valuable opportunity, it will contribute to the spread of S-FMR even further.

From the results of the present survey, the caries reduction rate of S-FMR in the permanent teeth was 36.6% for DMFT and 42.8% for DMFS, and person rates with DMF, DMFT, and DMFS were inhibited in both boys and girls. No gender differences were noted in the F+ group, but in the F− group, girls showed clearly higher values for all parameters, and in girls with more test teeth because of earlier eruption, the S-FMR effects were particularly high. The caries inhibitory effects of S-FMR on permanent teeth were equivalent to those found in other surveys [2, 7, 17]. S-FMR in elementary school appeared to promote the strengthening and remineralization of permanent teeth by fluoride application. In an examination of DMFT and DMFS by dentition of teeth, the values were about the same in the F+ and F− groups in the premolars and second molars that erupt from around the middle-years of elementary school, while the values in the F+ group were significantly lower than those in the F− group in the central and lateral incisors and the first molars that erupt in the lower-years of elementary school. Therefore, in the period of S-FMR participation, the effects of fluoride application were clear. As caries in the first molars accounted for about 85%, and first molar caries were more common in the mandible than in the maxilla, consideration should be given to measures against pit and fissure caries in combination with S-FMR [23, 24]. The subjects of this survey, based on S-FMR, also required pit and fissure sealing with the mandibular first molars given priority during their elementary school years.

As the presence of high-risk children was clear from the CO prevalence, it is necessary to provide appropriate after-care, in addition to S-FMR, in these children. The gender difference in CO was clearly eliminated under S-FMR management, but there were high-risk children with many CO teeth or CO tooth surfaces. Because of the pit and fissure caries in the first molars described previously, consideration should be given to pit and fissure sealing in addition to S-FMR [25–28]. Therefore, the use of CO as data for selection of subjects for preventive and control procedure is possible, but assessment by CO tooth number is simpler than that by CO tooth surface number. As S-FMR is the base in the F+ group, screening can be performed using the presence or absence of CO in the first molars as an index. In the F− group, subjects can be screened by totaling the number of CO teeth and using the children with the highest numbers of CO teeth (Figures 2 and 3).

## Figures and Tables

**Table 1:** Caries prevalence of 12-year-old children in two groups.

S-FMR experience in elementary school						Teeth				Tooth surfaces			
	Gender	Number	Mean number of erupted teeth	Mean number of erupted tooth surfaces	DMF person rate (%)	Mean DT	Mean MT	Mean FT	DMFT index*	Mean DT	Mean MT	Mean FT	DMFS index**
													
	Boys	319	25.2	114.3	44.5	0.08	0.00	1.18	1.27	0.18	0.00	1.98	2.16
F+	Girls	280	26.0	117.8	47.9	0.09	0.01	1.20	1.30	0.16	0.04	1.85	2.05
	Subtotal	599	25.6	116.0	46.1	0.09	0.00	1.19	1.28	0.17	0.02	1.92	2.05

	Boys	147	24.9	111.0	58.5	0.20	0.00	1.56	1.76	0.35	0.00	2.73	3.09
F−	Girls	135	25.8	116.4	71.9	0.27	0.00	2.04	2.31	0.53	0.00	3.82	4.36
	Subtotal	282	25.3	113.6	64.9	0.23	0.00	1.79	2.02	0.44	0.00	3.26	3.69

	Total	881	25.5	115.2	52.1	0.13	0.00	1.38	1.52	0.26	0.01	2.35	2.62

	Boys	—			46.5	—	—	—	1.30	—	—	—	—
Prefectural mean (2006)^†^	Girls	—			49.9	—	—	—	1.50	—	—	—	—
	Subtotal	—			48.1	—	—	—	1.40	—	—	—	—

	Boys	—			—	0.57	0.02	0.98	1.57	—	—	—	—
Nationwide mean (2006)^‡^	Girls	—			—	0.63	0.03	1.18	1.85	—	—	—	—
	Subtotal	—			—	0.60	0.03	1.08	1.71	—	—	—	—

*Average number of permanent teeth with dental caries (DT, MT, and FT).

**Average number of permanent tooth surfaces with dental caries (DS, MS, and FS).

^†^Results from Prefectural Survey on School Health Statistics (2006).

^‡^Results from National Survey on School Health Statistics by Ministry of Education, Culture, Sports, Science and Technology, Japan (2006).

**Table 2:** Caries prevalence of 12-year-old children by dentition in two groups.

S-FMR experience in elementary school			Mean DMFT				Mean DMFS			
	Gender	Number	12 11 21 22	16 26	15 14 24 25	17 27	12 11 21 22	16 26	15 14 24 25	17 27
			42 41 31 32	46 36	45 44 34 35	47 37	42 41 31 32	46 36	45 44 34 35	47 37
	Boys	319	0.03	1.13	0.06	0.04	0.08	1.93	0.09	0.05
F+	Girls	280	0.08	1.08	0.07	0.07	0.14	1.72	0.09	0.10
	Subtotal	599	0.06	1.11	0.06	0.06	0.11	1.83	0.09	0.07

	Boys	147	0.14	1.51	0.05	0.05	0.18	2.76	0.08	0.07
F−	Girls	135	0.21	1.92	0.10	0.08	0.44	3.64	0.15	0.12
	Subtotal	281	0.18	1.71	0.07	0.07	0.31	3.18	0.11	0.09
	Total	881	0.09	1.30	0.07	0.06	0.17	2.26	0.10	0.08

*11, 21, 31, 41: Central incisors; 12, 22, 32, 42: Lateral incisors; 16, 26, 36, 46: First molars; 14, 24, 34, 44: First premolars; 15, 25, 35, 45: Second premolars; 17, 27, 37, 47: Second molars.

**Table 3:** Prevalence of questionable caries under observation of permanent teeth in 12-year-old children in two groups.

S-FMR experience in elementary school				Teeth		Tooth surfaces	
	Gender	Number	CO* person rate (%)	Mean CO teeth	Percentage of CO teeth/total teeth (%)	Mean CO tooth surfaces	Percentage of CO tooth surfaces/total tooth surfaces (%)
	Boys	319	37.9	0.86	3.41	1.09	0.96
F+	Girls	280	39.6	0.92	3.53	1.15	0.98
	Subtotal	599	38.7	0.89	3.46	1.12	0.97
	Boys	147	55.0	1.35	5.41	3.31	2.98

F−	Girls	135	61.5	2.01	7.78	2.52	2.16
	Subtotal	282	57.8	1.66	6.56	2.93	2.58

	Total	881	44.8	1.14	4.45	1.70	1.48

*CO: questionable caries under observation.
